# Hybrid Assembly Improves Genome Quality and Completeness of *Trametes villosa* CCMB561 and Reveals a Huge Potential for Lignocellulose Breakdown

**DOI:** 10.3390/jof8020142

**Published:** 2022-01-30

**Authors:** Luiz Marcelo Ribeiro Tomé, Felipe Ferreira da Silva, Paula Luize Camargos Fonseca, Thairine Mendes-Pereira, Vasco Ariston de Carvalho Azevedo, Bertram Brenig, Fernanda Badotti, Aristóteles Góes-Neto

**Affiliations:** 1 Molecular and Computational Biology of Fungi Laboratory, Department of Microbiology, Instituto de Ciências Biológicas, Universidade Federal de Minas Gerais, Belo Horizonte 31270-901, MG, Brazil; marcelofsa_rt@hotmail.com (L.M.R.T.); felselva@gmail.com (F.F.d.S.); thairinemp@gmail.com (T.M.-P.); 2 Departamento de Genética, Ecologia e Evolução, Instituto de Ciências Biológicas, Universidade Federal de Minas Gerais, Belo Horizonte 31270-901, MG, Brazil; camargos.paulaluize@gmail.com; 3 Laboratório de Genética Celular e Molecular, Instituto de Ciências Biológicas, Universidade Federal de Minas Gerais, Belo Horizonte 31270-901, MG, Brazil; vascoariston@gmail.com; 4 Institute of Veterinary Medicine, Burckhardtweg, University of Göttingen, 37073 Göttingen, Germany; bbrenig@gwdg.de; 5 Department of Chemistry, Centro Federal de Educação Tecnológica de Minas Gerais, Belo Horizonte 30421-169, MG, Brazil; fbadotti@outlook.com

**Keywords:** *Trametes villosa* CCMB561, genome assembly, comparative genomics, lignocellulosic biomass, CAZymes

## Abstract

*Trametes villosa* is a wood-decaying fungus with great potential to be used in the bioconversion of agro-industrial residues and to obtain high-value-added products, such as biofuels. Nonetheless, the lack of high-quality genomic data hampers studies investigating genetic mechanisms and metabolic pathways in *T. villosa*, hindering its application in industry. Herein, applying a hybrid assembly pipeline using short reads (Illumina HiSeq) and long reads (Oxford Nanopore MinION), we obtained a high-quality genome for the *T. villosa* CCMB561 and investigated its genetic potential for lignocellulose breakdown. The new genome possesses 143 contigs, N50 of 1,009,271 bp, a total length of 46,748,415 bp, 14,540 protein-coding genes, 22 secondary metabolite gene clusters, and 426 genes encoding Carbohydrate-Active enzymes. Our CAZome annotation and comparative genomic analyses of nine *Trametes* spp. genomes revealed *T. villosa* CCMB561 as the species with the highest number of genes encoding lignin-modifying enzymes and a wide array of genes encoding proteins for the breakdown of cellulose, hemicellulose, and pectin. These results bring to light the potential of this isolate to be applied in the bioconversion of lignocellulose and will support future studies on the expression, regulation, and evolution of genes, proteins, and metabolic pathways regarding the bioconversion of lignocellulosic residues.

## 1. Introduction

Lignocellulosic biomass (LB), including agro-industrial residues, municipal solid wastes, and forest litter is one of the largest stocks of carbon and energy on Earth [1]. These are a sustainable, renewable, and abundant source of raw material, which can be (bio-) converted into high value-added products, such as bio-based chemicals, polymers, and fuels [1,2]. LB is composed mainly of cellulose (40–50%), hemicellulose (25–30%), and lignin (15–25%), as well as other compounds in lower proportions, such as pectin, proteins, extractables, and ash [3]. Although lignocellulosic materials are inexpensive, abundant, and considered a valuable feedstock for biorefineries (bio-based industry), a major challenge for their use is the degradation of lignin, which is a highly recalcitrant polymer, and the consecutive depolymerization of cellulose and hemicellulose (polysaccharides) to fermentable sugars (oligosaccharides and monosaccharides) [3,4,5]. Therefore, biomass needs to undergo physical, chemical, and/or biological pretreatment [4].

White-rot fungi (WRF) act efficiently in the biodegradation of plant biomass, possessing the metabolic machinery for the breakdown of all plant cell wall polymers (PCW) [6,7,8,9,10,11,12]. The depolymerization of the lignocellulose by WRF is carried out through the production and secretion of hydrolytic and oxidative enzymes belonging to glycoside hydrolases (GH), carbohydrate esterases (CE), pectate lyases (PL), and auxiliary activity oxidoreductases (AA) families, according to the classification in the Carbohydrate Active Enzymes database (CAZymes database) [13,14,15]. In addition to the PCW breakdown, WRF enzymes can also be applied in different industrial sectors, such as food, pulp and paper, textile, pharmaceutical, and biofuel, and be used in the bioremediation of polycyclic aromatic hydrocarbons (PAHs) and other xenobiotics [3,4,5,13,16,17].

Fungi of the genus *Trametes* (Polyporales, Basidiomycota) are classified as white-rot fungi and have the ability to simultaneously degrade all polymers of the lignocellulose [6,7,8,18,19]. In nature, they act as one of the major decomposers of wood and plant leaf litter, and play a central role in the carbon cycle, soil formation, and, consequently, in the maintenance of forest ecosystems [13,20,21]. To date, the genomes of the species *T. versicolor*, *T. coccinea*, *T. polyzona*, *T. hirsuta*, *T. cinnabarina*, *T. sanguinea*, and *T. pubescens*, among others, have already been sequenced, and research related to these genomes, as well as studies of transcriptomics and proteomics, described a set of mechanisms, genes, and metabolites involved in wood decay. Such knowledge has provided a huge aid toward the improvement in the production and industrial application of CAZymes [6,7,9,22,23,24,25,26,27,28].

*Trametes villosa* is an important species of the genus *Trametes*, and its potential to produce laccases, peroxidases, and cellulases in different lignocellulosic substrates has already been demonstrated at the small-scale biochemical level [29,30,31,32]. Regarding the strain *T. villosa* CCMB561, biochemical assays have shown its potential to produce ligninolytic enzymes using sugarcane bagasse as a substrate in different temperatures (from 20 °C to 33 °C) and pHs (from 4.60 to 9.38) [29,32]. Furthermore, Silva et al. (2014) obtained a reduction up to 63% in the lignin content of different agro-industrial wastes (sugarcane bagasse, sisal fiber, and coconut shell) using the enzyme Manganese Peroxidase (MnP) produced by *T. villosa* CCMB561 [32]. Despite the potential of this isolate for biotechnological applications, so far, no genomic study has been carried out to comprehensively understand the genetic repertoire and mechanisms involved in the complex breakdown of all plant cell wall components (lignin, hemicellulose, cellulose, and pectin).

Indeed, the genome of *T. villosa* CCMB561 has previously been sequenced and a public, preliminary draft version is available in the NCBI database (GenBank accession: GCA_002964805.1) [24]. Nevertheless, the draft genome has a high fragmentation rate (10,327 contigs), high duplication of single-copy ortholog genes, and a genome size larger than expected for the genus [24]. The low quality of these data limits downstream analysis, such as the identification, characterization, and understanding of the expression and regulation of genes and proteins.

Therefore, in order to obtain a high-quality genome and then contribute to further studies aimed at understanding the genetic bases of lignocellulose breakdown by the WRF, we have sequenced the genome of the isolate *T. villosa* CCMB561 jointly using second- (HiSeq 2500—Illumina) and third-generation (MinION—Oxford Nanopore) sequencing platforms. Different assembly strategies were tested and are described in this study. Coding regions, transfer RNAs (tRNAs), transposable elements, and CAZymes genes were annotated. Furthermore, comparative and phylogenomic analysis including genomes of other isolates of the genus *Trametes* were performed. Our findings revealed that using a hybrid assembly approach (a combination of short- and long-read sequencing from distinct platforms), it was possible to acquire a genome with much better completeness and contiguity when compared to the draft genome available. The new assembled genome presented 143 contigs, a size of 46.748 Mb, 14,540 proteins-encoding genes, and 22 secondary metabolite gene clusters (SMGCs). In addition, a wide array of genes encoding lignocellulose-modifying enzymes was identified, revealing a huge potential of the isolate *T. villosa* CCMB561 to act in the degradation of all lignocellulose polymers, making it a high-potential strain to be industrially used. 

## 2. Materials and Methods

### 2.1. Fungal Strain and Extraction of Genomic DNA

The fungal strain *T. villosa* CCMB561 was isolated from field-collected basidiomata growing on a decaying tree branch (unidentified angiosperm) in the semiarid region of northeastern Brazil (Serra das Candeias, Quijingue, Bahia, Brazil; Lat: 39°04′30″ W and Long: 10°55′16″ S). Dehydrated basidiomata were deposited in the HUEFS herbarium (HUEFS108280), and the culture derived from the basidiomata tissue was preserved in sterile distilled water and deposited in the Culture Collection of Microorganisms of Bahia (CCMB, Feira de Santana, Bahia, Brazil) under access code CCMB561. The isolate was grown on Malt Extract Agar (2% Malt Extract, 2% dextrose and 2% Agar) at 28 ± 2 °C for seven days. Then, the total DNA was extracted using the ZymoBIOMICS^TM^ DNA Miniprep Kit (Zymo Research, Irvine, CA, USA). The DNA sample was analyzed qualitatively by agarose gel electrophoresis 1%, and quantitatively by a Nanodrop 1000 ND spectrophotometer (Thermo Scientific, Waltham, MA, USA) and Qubit fluorometer (Invitrogen, Waltham, MA, USA). For species-level certification of the extracted DNA, the internal transcribed region (ITS1-5.8S-ITS2) was amplified and sequenced using the ITS 6 (5′-TTCCCGCTTCACTCGCAGT-3′) and ITS 8 (5′-AGTCGTAACAAGGTTTCCGTAGGTG-3′) primers [33]. Amplification reaction, purification, and sequencing of the amplicons were carried out according to the methods described by Tomé et al. 2019 [34]. 

### 2.2. MinION Library Preparation and Sequencing

We fragmented genomic DNA [8 μg] to approximately 8 Kbp using the Covaris g-TUBE (Covaris, Woburn, MA, USA). After fragmentation, 1200 ng of DNA was purified using the AMPureXP beads (Beckman Coulter Inc., Brea, CA, USA), not adopting the DNA repair step. The sequencing library was prepared using the Ligation Sequencing Kit 1D (SQK-LSK108), the Native Barcoding Kit 1D (EXP-NBD103), and the Library Loading Bead Kit (EXP-LLB001), following the recommendations of Oxford Nanopore Technologies. The library was sequenced for 48 h in the flowcell FLO-MIN106 (ID: FAK07371) using the MinKNOW program with the real-time base calling function enabled. Porechop software (https://github.com/rrwick/Porechop, accessed on 15 January 2020) was used to demultiplex the libraries and remove the adapters (Figure S1).

### 2.3. Illumina Library Preparation and Sequencing

The sequencing library was prepared from genomic DNA [1 μg] using the NEBNext Fast DNA Fragmentation and Library Preparation Kit (New England Biolabs, Ipswich, MA, USA) following the manufacturer’s recommendations. The library quality was assessed using the Agilent 2100 Bioanalyzer equipment, and the paired-end DNA sequencing was carried out in the Illumina HiSeq 2500 platform. After sequencing, the raw reads quality was assessed using the FastQC v0.11.5 software (https://github.com/s-andrews/FastQC, accessed on 15 January 2020). Adapter sequences and bases with low quality (Phred score <20) were trimmed using BBDuk software (https://sourceforge.net/projects/bbmap/, accessed on 15 January 2020) (Figure S1). Genome features such as size, heterozygosity, and repetitiveness were assessed prior to genome assembly using Jellyfish and GenomeScope 2.0 [35,36].

### 2.4. De Novo Genome Assembly and Assessment

The genome assembly was carried out using different approaches and software (Figure S1). MinION long-reads were assembled using (i) Flye [37], (ii) Canu [38], (iii) Racon [39], and the (iv) CANU-smartdenovo pipeline with default parameters [40] while Illumina short reads were assembled using the (v) MaSuRCA software with default parameters [41]. Hybrid assemblies using Illumina and MinION reads were performed using the software (vi) MaSuRCA, (vii) SPAdes [42], and the assembly workflow (viii) MaSuRCA-Purge_dups [41,43]. Genome quality and completeness for each assembly were evaluated using QUAST v4.6.0 [44] and BUSCO v4 (Benchmarking Universal Single-Copy Orthologs) [45]. BUSCO analyses were performed using the database basidiomycota_odb10. 

### 2.5. Genome Annotation and Gene Ontology Analyses

Genome annotation was performed using the MAKER2 v2.31.9 software [46,47,48] and the following ab initio gene prediction software: SNAP [49], Augustus [50], and GeneMark [51] (Figure S2). Low- and high-complexity repetitive genomic regions were masked using the RepeatMasker [52], the Repbase database, and the RepeatRunner software [48]. The identification of gene regions and the prediction of proteins were performed through the alignment of ESTs (Expressed Sequence Tags) and proteins of the genus *Trametes* (obtained from NCBI until September 2020) using the BLAST algorithm and the Exonerate program. After the annotation by evidence, the software SNAP, Augustus, and GeneMark were used in the further annotation steps. The annotation metrics, such as the number of genes, exons, and introns, were obtained using GAG software (Genome Annotation Generator) [53]. The assignment of gene function was carried out with the support of tools provided by MAKER [47], the makeblastdb application, the UniProt database (uniprot_sprot.fasta), and the blastp algorithm. Gene ontology analyses were carried out in the web server GoFeat (Gene Ontology Functional Enrichment Annotation Tool), with the support of the following databases: Uniprot, NCBI protein, KEGG, InterPro, Pfam, EMBL, and Gene Ontology [54]. The prediction of transfer RNA (tRNA) was performed using the software tRNAscan-SE [55]. Secondary metabolite gene clusters (SMGCs) were predicted using the online tool antiSMASH 6.0.1 [56].

### 2.6. Repeat Annotation

Transposable elements (TE) were identified de novo using the RepeatModeler package (repeatmasker.org/RepeatModeler, accessed on 15 September 2020) with the support of RepeatMasker [52], RECON [57], RepeatScout [58], TRF [59], and RMBlast. The obtained TE library (consensus sequences) was filtered by removing all sequences <100 bp and those showing significant hits with proteins not identified as TE using blastx and the UniProt database [7]. The classification and number of occurrences of TE were assessed using the RepeatMasker tool. 

### 2.7. Comparative Genomics and Phylogenomics

The genome of the fungus *T. villosa* CCMB561 was compared with the following seven genomes publicly available at the National Center for Biotechnology Information (NCBI, https://www.ncbi.nlm.nih.gov/, accessed on 15 March 2020): *T. coccinea* (GCA_002092935.1), *T. sanguinea* (GCA_008973685.1), *T. cinnabarina* (GCA_000765035.1), *T. hirsuta* (GCA_001302255.2), *T. polyzona* (GCA_001939255.1), *T. pubescens* (GCA_001895945.1), and *T. versicolor* (GCF_000271585.1); and two publicly available at the Joint Genome Institute (JGI, https://genome.jgi.doe.gov/portal/, accessed on 15 March 2020): *T. ljubarskyi* (CIRM1659) and *T. elegans* (CIRM1663, synonym: *Artolenzites elegans*). The completeness and the main metrics of the retrieved genomes were assessed using BUSCO and QUAST, respectively. In order to standardize and improve the accuracy of the comparative analyses, all genomes were reannotated, and the transposable elements were identified using the methods described in Section 2.5 and Section 2.6, respectively. Phylogenomic analyses were carried out using the script BUSCO_phylogenomics (https://github.com/jamiemcg/BUSCO_phylogenomics, accessed on 15 October 2020), in which single-copy ortholog genes were aligned using MUSCLE. The alignment was trimmed using trimAl [60], and the estimation of the best-fit model was performed using ModelFinder. The maximum likelihood phylogenetic tree was generated using the IQ-TREE software, adopting the supermatrix method [61]. The consensus tree was constructed considering 1000 bootstrap replicates and visualized in FigTree v1.4.3. The species *Polyporus brumalis* (Polyporales, Basidiomycota—GCA_001792895.1) was used in the phylogenomic analyses as the outgroup. Network and correlation analyses were performed based on the genome length, number of genes, TE coverage, GC content, and number of tRNAs, using the PAST 4.04 software. The Bray–Curtis dissimilarity index (edge cutoff: 50%) was adopted for the network analyses while the correlation analyses were conducted using Pearson’s correlation.

### 2.8. CAZy Annotation and Potential for Lignocellulose Degradation

The Carbohydrate Active enzymes (CAZymes) of the *Trametes* species were functionally annotated using the dbCAN2 web server (http://bcb.unl.edu/dbCAN2/, accessed on 15 November 2020) with the integration of the following automated annotation tools/databases: (i) HMMER, (ii) DIAMOND, and (iii) Hotpep [62]. The dbCAN outputs were manually curated. Proteins identified by two or three tools (HMMER, DIAMOND, and HOTPEP) were considered correctly classified while those identified by only one tool were subjected to blastp analyses (protein–protein BLAST) to confirm the dbCAN classification. After annotation and manual curation, proteins related to cellulose, hemicellulose, lignin, and pectin degradation were counted and heat maps were generated using the pheatmap package (1.0.12) in R software (R 4.0.3). 

## 3. Results and Discussion

### 3.1. Illumina and MinION Sequencing

After adapter removal and quality trimming, 48,347,940 short reads were obtained through the Illumina sequencing (read length about 150 pb), corresponding to approximately 14 Gb (Table 1). Using these data and the GenomeScope software, the genome of the isolate *T. villosa* CCMB561 was estimated to have 44,895,640 bp in length, a homozygosity rate of 97.4%, and heterozygosity of 2.6% (Supplementary Data S1). The sequencing using the Oxford Nanopore platform generated 1,043,247 long reads, totaling 8.1 Gb. The long reads had an average size of 4.47 kb, N50 of 5.1 kb, and the longest read with 21,613 bp (Table 1). According to the estimated genome size of the CCMB561 strain, coverages of 129× and 93× were obtained through sequencing on the Illumina and Oxford Nanopore platforms, respectively. In previous studies, genomes with lengths similar to the estimated length of *T. villosa* CCMB561 were assembled with good contiguity and completeness using even smaller coverages than those obtained in this study. For example, the genome of the fungus *Leptosphaeria maculans* Nz-T4, which has a size of 43.42 Mb, was assembled in 288 contigs, using 56× long reads and 98× short reads [63]. In another study, the genome of the alga *Chlorella variabilis,* with a size of 46.67 Mb, was assembled into 302 contigs, using 56× long reads and 78× short reads [64]. Thus, the sequencing coverage obtained in this study was considered sufficient for the high-quality assembly of the genome of *T. villosa* CCMB561.

### 3.2. Genome Assembly and Assessment

**Figure 1:**
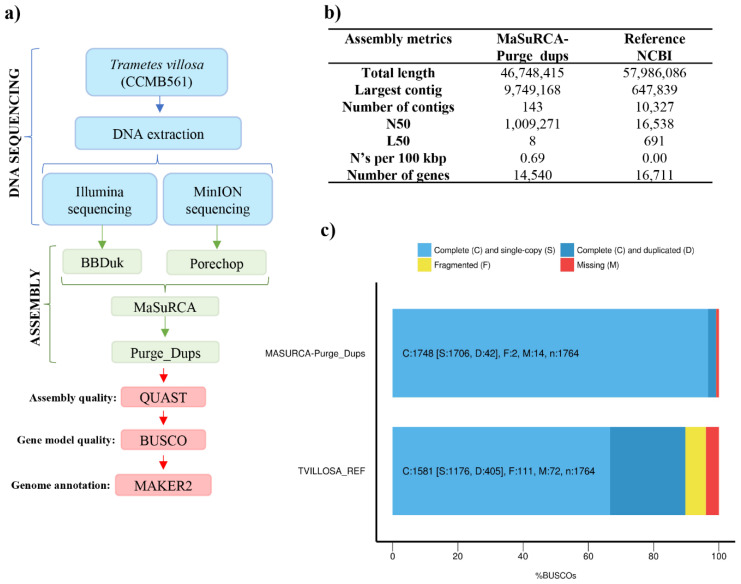
Overview of the newly assembled genome of *Trametes villosa* CCMB561. (**a**) Assembly workflow proposed as the best approach for genome assembly. (**b**) Summary evaluation of the genome assembled through MaSuRCa-Purge_Dups workflow and the reference genome of *Trametes villosa* deposited in the NCBI database (GCA_002964805.1). (**c**) BUSCO completeness assessment of the new genome and the reference of *Trametes villosa* previously deposited in the NCBI (GCA_002964805.1).

In this study, eight assembly strategies were tested: one using exclusively short reads (Illumina HiSeq), four using only long reads (Oxford Nanopore MinION), and three based on the hybrid assembly, combining short and long reads (Table 2). The best result was obtained using the assembly workflow MaSuRCa-Purge_Dups (Hybrid assembly) (Table 2 and Figure 1a), which used MaSuRCa software to generate a primary assembly and the Purge_Dups program to identify and remove haplotypic duplications. The use of this workflow resulted in a genome with 143 contigs, a total length of 46,748,415 bp, the largest contig with 9,749,168 bp, and N50 of 1,009,271 bp (Table 2). This genome had the smallest difference according to the genome size estimated by GenomeScope2 (difference of 1,852,775 bp) and presented the best completeness index through the BUSCO analysis. The assembled genome presented 99.1% of the orthologous genes searched, of which 96.7% are single copies, 2.4% are duplicated, and 0.1% are fragmented. Finally, only 0.8% of the genes were not found (Table 3). 

The assemblies in which the Purge_Dups software was not used showed a high degree of gene duplication and/or genome size greater than expected for the *Trametes* genus (~44 Mb), except when the CANU-smartdenovo pipeline was used (Table 2 and Table 3). Previous studies have already demonstrated that, in order to facilitate the genome sequencing and assembly from dikaryotic fungi, dedikaryotization and thus the obtainment of a monokaryotic isolate is an essential step [27]. Hence, the high rate of gene duplication and genome size larger than expected may be related to the sequencing of the dikaryotic mycelium. Nevertheless, the use of Purge_Dups software [43] allowed us to remove duplications and increase genome contiguity without the need to obtain a monokaryotic isolate, which is laborious and time-consuming. 

A high degree of fragmentation was detected when the genome was assembled using only Illumina sequencing data (Table 2). Conversely, when only long reads were used, the genome showed low fragmentation, but smaller completeness (Table 2 and Table 3). This result is due to Illumina HiSeq sequencing generating reads with sizes between 100 and 250 bp, which leads to greater fragmentation of the genome. On the other hand, the MinION approach, despite generating long reads, which could exceed 2 Mb, presents a higher error rate (1D sequencing), which may imply a lower completeness rate [65]. 

Compared to the preliminary and draft genome of *T. villosa* CCMB561 deposited at the NCBI, herein, using the MaSuRCa-Purge_Dups assembly workflow (Figure 1a), we obtained a much higher-quality genome, with a significant reduction in the number of contigs (from 10,327 to 143) and improvement in the metrics N50, L50, and size of the largest contig (Figure 1b). Furthermore, based on the BUSCO analysis (Figure 1c), the newly assembled genome has better completeness indices and fewer duplicated, fragmented, and missing genes. Similarly, Maggiori et al. (2021) demonstrated that by using a hybrid assembly approach (HiSeq + MinION), it was possible to obtain a greater number of genomes from metagenomic samples, with longer contigs, more coding sequences, higher completeness, less contamination, and higher N50 [66].

### 3.3. Genome Annotation and Gene Ontology (GO) Analysis

The annotation results showed that *T. villosa* CCMB561 has 14,540 protein-coding genes, 86,516 exons, and 71,976 introns, which correspond to 66% of the genome (Table S1). Each gene had, on average, six exons and five introns with sizes of 265 bp and 112 bp, respectively, which agrees with other species of the genus (Table S1). In total, 274 transposable elements (TEs) were identified in the CCMB561 isolate, corresponding to 7.13% of the genome. The number of transport RNA (tRNAs) identified was 334.

**Figure 2:**
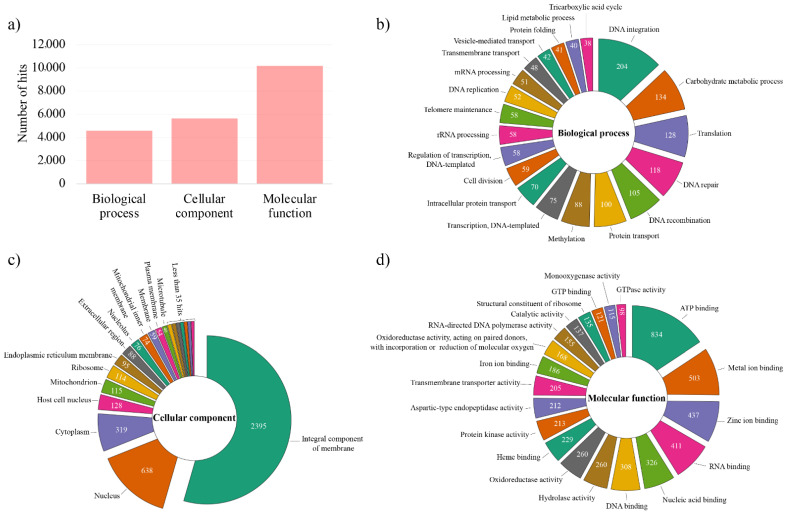
Gene Ontology (GO) functional annotation of *Trametes villosa* CCMB561 proteins. (**a**) Number of hits (GO terms) associated with the predicted proteins by GO categories (Biological process, Cellular component, and Molecular function), in which one protein can be associated with multiple GO terms. (**b**–**d**) The 20 most assigned terms per category in the GO enrichment analysis.

According to gene ontology (GO) analysis, 8169 proteins of *T. villosa* CCMB561 were associated with GO terms, corresponding to 56.18% of the predicted sequences. Functionally annotated proteins were classified into three categories: (i) molecular function, (ii) cellular component, and (iii) biological process (Figure 2). In the “cellular component” category (Figure 2c), most proteins were associated with the terms “integral component of membrane” (2395), “nucleus” (638), and “cytoplasm” (319), which are terms related to the cell anatomy.

In the categories “biological processes” (Figure 2b) and “molecular function” (Figure 2d), terms related to the degradation of lignocellulosic biomass, such as “carbohydrate metabolic process” (GO:0005975), “hydrolase activity” (GO:0016787) “oxidoreductase activity” (GO:0016491), and “heme-binding” (GO:0020037) are among the most representative. This is an expected result since species from the genus *Trametes* act in the degradation of the main components of lignocellulosic biomass through the production and secretion of a large set of hydrolytic and oxidative enzymes [7,9,12,67]. Additionally, 205 proteins associated with “transmembrane transporter activity” (GO:0022857) have been identified (Figure 2d). This term could indicate enzymes that are secreted and have extracellular activity, such as lignocellulose-degrading enzymes [6].

In the genome of the CCMB561 strain, 147 cytochrome P450 (CYP) genes and one NADPH-cytochrome P450 reductase (CPR) gene were identified. Similar results were found by Sun et al. (2018), who identified in *T. versicolor* the presence of only one NADPH gene-cytochrome P450 reductase and multiple sequences belonging to genes of the cytochrome P450 family [68]. These genes are widely known for their importance in the degradation of lignin and organic pollutants (aromatic and xenobiotic compounds) [68]. Genes from the CYP family also play a role in the metabolism and adaptation of fungi to specific ecological niches [6,69]. Complementarily, Liu et al. (2019) described that the fungus *Trametes trogii* S0301 has 158 CYPs that may be related to a variety of metabolic functions [6].

### 3.4. Annotation of Secondary Metabolite Gene Clusters (SMGCs) and CAZymes of Trametes Villosa CCMB561

#### 3.4.1. Secondary Metabolite Gene Clusters (SMGCs)

Fungi possess many gene clusters responsible for producing Secondary Metabolites (SMs), which have important ecological functions [70]. SMs are not essential for the normal growth of the organism but may act as defense compounds (e.g., against fungi and bacteria) and signaling molecules, being fundamental for ecological interactions and survival [71]. Because of their bioactive pharmacological properties, these molecules have been widely studied and tested in the healthcare industries to be used as antibiotics, antifungals, anti-inflammatory, and anticancer agents [71,72].

**Figure 3:**
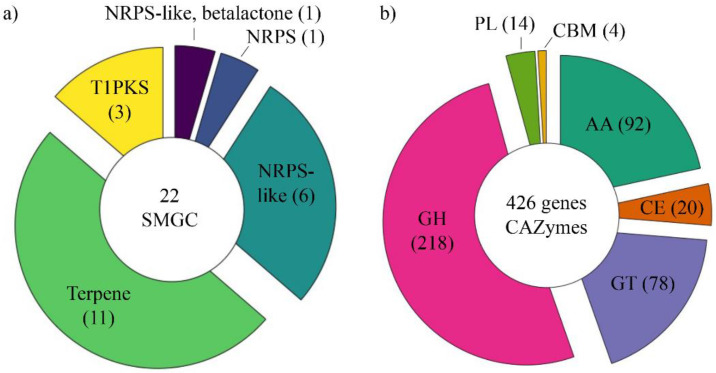
Annotation of Secondary metabolite gene clusters (SMGCs) and Carbohydrate-Active enzymes (CAZymes). (**a**) SMGCs identified in the genome of *Trametes villosa* CCMB561. (**b**) CAZymes identified in the genome of *Trametes villosa* CCMB561.

In *T. villosa* CCMB561, we identified 22 SM biosynthesis clusters, which comprise one non-ribosomal peptide synthetase (NRPS) cluster, one NRPS-like/betalactone cluster, three Type I Polyketide synthase (T1PKS) clusters, six NRPS-like clusters, and eleven Terpene clusters (Figure 3a and Supplementary Data S2). The NRPS-type cluster (Region 33.1/scf7180000000809) was identified with 100% of similarity with the basidioferrin compound cluster from *Gelatoporia subvermispora* (BGC0001527.1) (Polyporales, Basidiomycota). Basidioferrin is widely distributed in basidiomycetes and is part of the siderophore synthetases family, which are enzymes responsible for the biosynthesis of siderophores and iron metabolism [73]. Previous studies have reported that in most bacteria and fungi (pathogenic and non-pathogenic), the acquisition of high-affinity iron is mediated by siderophore-dependent pathways [74].

Most of the secondary metabolites biosynthesis clusters identified in *T. villosa* CCMB561 were assigned to the terpene type (11 clusters) (Figure 3a). Terpenoids have multiple biological activities and comprise sesquiterpenoids, diterpenoids, and triterpenoids. Their activities in inducing the apoptosis of human tumor cells, antibacterial, antimetastasis, and anti-HIV activity have already been demonstrated [72]. Using the MIBiG database (Minimum Information about a Biosynthetic Gene cluster), whole or partial genes of some known terpene clusters were identified. Our results showed that fragments of the clusters squalestatin S1 from *Aspergillus sp.* (BGC0001839.1), geosmin from *Streptomyces coelicolor* (BGC0001181.1), and koraiol from *Fusarium fujikuroi* (BGC0001642.1) were identified in the CCMB561 genome.

It is important to highlight that most clusters (21 clusters), despite being classified according to the type, had no similarity in the MIBiG database with the biosynthesis cluster of known compounds. Therefore, the CCMB561 isolate has the potential to produce a variety of secondary metabolites; however, these metabolites have not yet been identified or reported in the literature.

#### 3.4.2. CAZome Annotation

The CAZome annotation results demonstrated that *T. villosa* CCMB561 possesses 426 genes encoding CAZymes, comprising 218 glycoside hydrolases (GH), 20 carbohydrate esterases (CE), 78 glycosyltransferases (GT), 14 polysaccharide lyases (PL), 4 Carbohydrate-binding modules (CBM), and 92 auxiliary activity enzymes (AA) (Figure 3b and Supplementary Data S3). Among the families that act in the depolymerization of lignocellulose, glycoside hydrolases (GHs) include glycosidases with activity in the hydrolysis of glycosidic bonds between two or more carbohydrates [14,15]. Carbohydrate esterases (CEs) act by removing ester-based modifications in polysaccharides, facilitating the action of GHs [14,15]. Polysaccharide Lyases (PLs) cleave glycosidic bonds from uronic acid-containing polysaccharides (e.g., pectin) [14,15]. Finally, the auxiliary activity families (AAs) include oxidative enzymes that act mainly in the depolymerization of lignin, helping the enzymes from GH, CE, and PL classes to gain access to carbohydrates from the plant cell wall [75].

The Carbohydrate-binding module (CBM) domain was detected in 33 genes encoding enzymes belonging to classes AA, GH, and CE (Supplementary Data S3). Most of the CBM domains (18 in total) found in the annotated genes belong to the Carbohydrate-binding module family 1 (CBM1) and were found in enzyme-encoding genes acting in the cellulose (AA9, GH3, GH5_5, GH6, and GH131 families) and hemicellulose breakdown (CE1, CE15, GH5_7, GH10, and GH74 families). CBM domains promote an enzyme association with the substrate and increase enzymatic hydrolysis and degradation of polysaccharides [76,77]. In addition, four dye-decolorizing peroxidases (DyPs) with the potential to oxidize lignin-like compounds and other phenolic polymers were identified in the genome of the isolate *T. villosa* CCMB561 [10].

Similar results of our CAZome annotation have been reported for the species *T. versicolor*, which has 424 genes encoding Carbohydrate-Active enzymes (CAZymes) [18]. *T. versicolor* is closely related to *T. villosa* and is one of the most common and widespread species of white-rot and basidiomata-forming fungi, showing great potential to act in lignocellulose breakdown [7,18].

### 3.5. Comparative Genomics and Phylogenomics of the Genus Trametes

**Figure 4:**
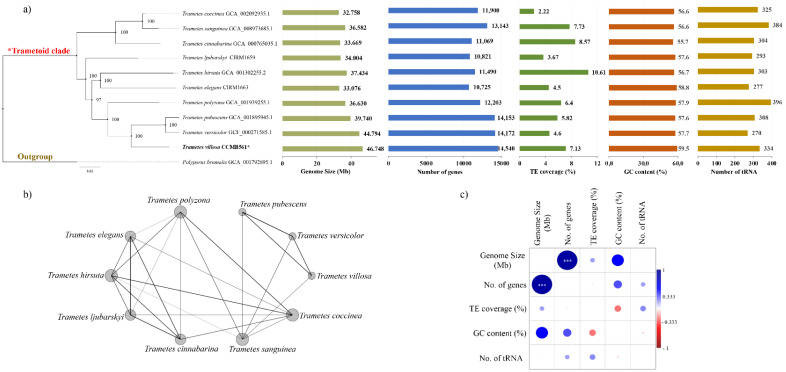
Comparative genomics results overview. (**a**) Maximum-likelihood phylogenomic tree constructed using the newly assembled genome of *Trametes villosa* CCMB561 (marked with *) and nine available genomes from the *Trametes* genus. Bootstrap values are expressed in percentage and the features of each genome are shown beside the phylogeny. (**b**) Network plot created using a matrix containing the values of genome size, number of genes, TE coverage, GC content, and number of tRNA of each genome. (**c**) Correlation analysis among the main metrics of the genome (statistically significant correlations are represented with *).

The Maximum Likelihood phylogenetic matrix (RaxML) included 11 sequences with 798,158 amino acids from 1346 concatenate proteins of each genome. From these amino acids, 146,797 had distinct patterns, 152,144 were parsimoniously informative, 128,942 were parsimoniously non-informative, and 517,072 were constant characters. According to the tree topology (Figure 4a), *T. villosa*, *T. versicolor,* and *T. pubescens* were grouped in the same clade with a 100% bootstrap while the monophyletic clade formed by *T. coccinea*, *T. sanguinea,* and *T. cinnabarina* is the most phylogenetically distant from *Trametes villosa* CCMB561. These clustering patterns have already been reported in a previous phylogenetic reconstruction of Polyporales fungi, based on LSU and ITS ribosomal DNA markers [78]. In the Complex network analysis (Figure 4b), as well as in the phylogenomic analysis, *T. villosa*, *T. versicolor*, and *T. pubescens* were grouped together, suggesting these species have similar structural genomic characteristics, reinforcing the phylogenetic proximity.

The fungal genome size can be impacted by different factors and is directly related to the fungal lifestyle, as well as adaptive and ecological needs [79,80,81]. Therefore, herein we evaluated the genome size of *Trametes* spp. and features that may impact the genome length, such as the number of genes, number and coverage of TEs, number of tRNAs, and GC content. Among the species analyzed, the genome sizes ranged from 32.758 Mb (*T. coccinea*) to 46.748 Mb (*T. villosa* CCMB561), and the number of genes from 10,725 (*T. elegans*) to 14,540 (*T. villosa* CCMB561) (Figure 4a). The average genome length for Basidiomycetes is 46.48 Mb, ranging from 9.82 (*Wallemia sebi*) to 130.65 Mb (*Dendrothele bispora*) [79]. The genomes of the *Trametes* spp. evaluated in this study had sizes within this range, and *T. villosa* CCMB561 was the species with a genome size closest to the mean described to the phylum Basidiomycota. This species also presented the highest number of genes, followed by the closely related *T. versicolor* and *T. pubescens*.

The coverage of TEs in the genomes ranged from 2.22% (*T. coccinea*) to 10.61% (*T. hirsuta*) (Figure 4a). Most of the classified TEs were LTR retrotransposons (long terminal repeats) belonging to Copia and/or Gypsy types (Table 4). Moreover, retrotransposons SINEs and LINEs, as well as DNA transposons and Helitrons were also identified. TEs can act in the modulation of genomes, through recombination and transposition, which can lead to chromosomal rearrangements and alter gene expression [80]. In Basidiomycota, the genome content corresponding to TEs can vary from 0.1 to 45.2% (average around 11%) and, usually, most of the TEs are LTR retrotransposons (Gypsy and Copia) [82]. A possible reason for the high copy number of TEs class I in Basidiomycota, including the studied species, is its transposition mechanism, which uses an intermediate RNA, resulting in the increased proliferative success of the TEs [82].

The GC content had no significant variation among the analyzed species and ranged from 55.7% (*T. cinnabarina*) to 59.5% (*T. villosa*) (Figure 4a). On the other hand, the number of tRNAs varied from 270 (*T. versicolor*) to 396 (*T. polyzona*), with a difference of more than 100 tRNAs among the species in this genus (Figure 4a). Transfer RNAs play a central role in protein biosynthesis and are involved in many biological functions in eukaryotic organisms, such as in the regulation of gene expression [83]. Therefore, this difference/expansion in the number of tRNAs could be related to specific evolutionary mechanisms and the lifestyle of each species [84].

Genome size and the number of genes were the only features that had a positive and statistically significant correlation (*p* < 0.05) in the genomes of *Trametes* spp. (Figure 4c). Interestingly, TE coverage was not significantly correlated with genome size. Similar results were described by Castanera et al. (2017), who described for some genera of the Agaricomycotina subphylum, including *Trametes* species, a high correlation between genome size and gene content while the correlation between the number of TEs and genome size was unclear [82].

Overall, all the analyzed *Trametes* genomes displayed variability in relation to the analyzed features, with no clear pattern concerning the genome size, number of genes, number and coverage of TEs, and number of tRNAs. This variability may be related to the environment, selective pressures, and ecological and evolutionary factors to which each species is subjected to [84].

### 3.6. Potential for Lignocellulose Breakdown by Trametes spp.

**Figure 5:**
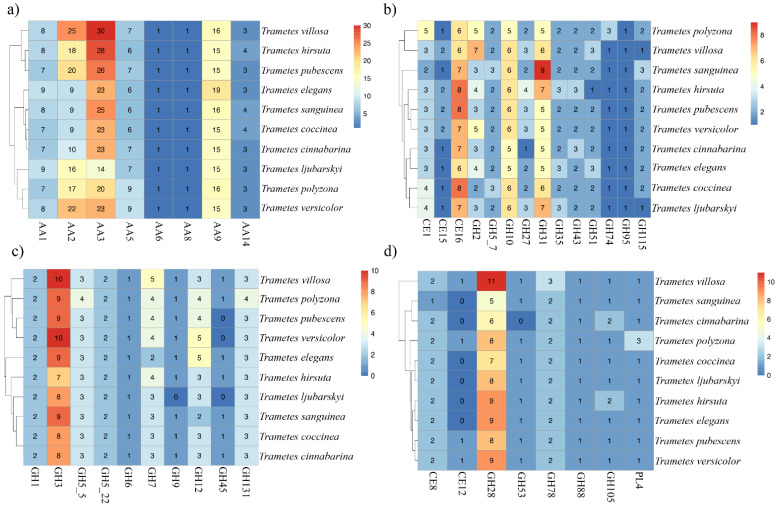
CAZyme-encoding genes involved in the degradation of lignocellulosic biomass. (**a**) Number of auxiliary redox enzyme-encoding genes. (**b**) Number of hemicellulose-degrading enzyme-encoding genes. (**c**) Number of cellulose breakdown enzyme-encoding genes. (**d**) Number of pectin-degrading enzyme-encoding genes.

Exploring the presence, abundance, and composition of oxidoreductases and carbohydrate-active enzymes (CAZymes) in wood-decay fungi provides important information on its nutritional preferences and adaptations, the metabolic pathways used, as well as the expansion and evolution of gene families related to lignocellulose breakdown. This information is important for an understanding of fungal biology and further application in the biotechnology industry. In this study, ten genomes of different *Trametes* species were evaluated for the presence of 40 gene families encoding enzymes that act in the breakdown of lignin, hemicellulose, cellulose, and pectin (Figure 5).

The lignin degradation is mainly performed by white-rot fungi and involves a series of enzymes classified as an Auxiliary Activity family (AA) [7,10,27,85]. In *Trametes* spp. genomes, many AAs were identified and have a central role in lignin modification (Figure 5a). The studied genomes harbor from 7 to 9 AA1-encoding genes, and in *T. villosa*, eight genes were recognized as AA1 (Figure 5a). The AA1 family encompasses multicopper oxidases (Laccases) that act directly on a wide range of aromatic and phenolic compounds, such as lignin [75,85,86]. These enzymes do not require cofactors for their activity, so they are of great interest for industrial applications [86].

The greatest number of lignin-modifying enzymes encoding genes was identified in families AA2 (9–25 genes) and AA3 (14–30 genes) (Figure 5a). The AA2 family includes Lignin Peroxidase (LiP), Manganese Peroxidase (MnP), and Versatile Peroxidase (VP). These enzymes are classified as class II peroxidases (PODs), since they use hydrogen peroxide (H_2_O_2_) as a cofactor for lignin breakdown [7,75,85,86]. The results displayed in Figure 5a also demonstrated an expansion in the number of AA2-encoding genes in *T. villosa* (25 copies), *T. hirsuta* (18 copies), *T. pubescens* (20 copies), *T. ljubarskyi* (16 copies), *T. polyzona* (17 copies), and *T. versicolor* (22 copies). This result could indicate a possible metabolic adaptation of these species to initially use lignin as the main carbon source for their growth. It is worth noting that *T. villosa* was the species with the largest number of AA2-encoding genes.

In the metabolic pathway of lignin degradation, enzymes belonging to the AA3 and AA5 families play a fundamental role in the generation of H_2_O_2_ and activation of PODs [75,86]. AA3 are flavoproteins containing a flavin-adenine dinucleotide (FAD)-binding domain and are generally recognized as glucose-methanol-choline (GMC) oxidoreductases, which include Pyranose 2-oxidase, Alcohol oxidase, Glucose 1-oxidase, and Aryl-alcohol oxidase [75,86]. In our analyses, AA3 was the family with the highest number of identified genes, ranging from 14 to 30 copies per species. Additionally, *T. villosa* was the species with the highest number of AA3-encoding genes, possessing 30 copies.

The AA5 family is composed of copper radical oxidases and includes two described subfamilies: AA5_1 (glyoxal oxidase) and AA5_2 (galactose oxidases) [75,86]. The *Trametes* spp. genomes harbor 6 to 9 AA5-encoding genes, and in *T. villosa*, seven AA5 genes were identified. One AA6-encoding gene was conserved in all *Trametes* species. This enzymatic family includes 1,4-benzoquinone reductases, which are responsible for the intracellular cleavage of aromatic compounds and for the protection of fungal cells from reactive quinone compounds [75].

Regarding the families of hydrolytic enzymes with activity in the depolymerization of hemicellulose, CE16 (acetylesterases—six to eight copies), GH10 (endo-1,4-β-xylanases—five to six copies), and GH31 (alpha/beta-glucosidases/α-xylosidases—five to nine copies) were the families with the highest number of genes (Figure 5b). Carbohydrate Esterase family 16 (acetylesterase activity) plays a fundamental role in the deacetylation of hemicellulose units, allowing the activity of glycoside hydrolases [77]. The GH10 family includes endoxylanases (endo-β-1,4-endoxylanases) that act on the degradation of linear chains of β-1,4-linked D-xylose residues [3,18,77]. The CAZy family GH31 mainly includes enzymes with α-glucosidase activity [77]. Besides, other xylanases and xyloglucanases belonging to the families GH5_7 (β-1,4-endoxylanases/β-1,4-endoglucanases), GH51 (β-1,4-endoxylanases), GH43 (β-xylosidase), GH35 (β-galactosidases), and GH74 (endoglucanases) were also identified in the analyzed genomes (Figure 5b).

Xylan-type hemicellulose can also be degraded through the action of oxidative enzymes belonging to the AA14 family, which include copper-dependent lytic polysaccharide monooxygenases (LPMOs) [87]. In the evaluated *Trametes* spp. genomes, three to four genes encoding lytic xylan monooxygenase were identified (Figure 5a). Different families were annotated in the analyzed genomes responsible for the degradation of hemicellulose side chains, such as GH2 (β-galactosidases), GH27 (α-galactosidase), GH95 (α-L-fucosidase), GH115 (xylan α-1,2-glucuronidase), CE1 (acetyl xylan esterase, EC 3.1.1.72), and CE15 (glucuronoyl esterase) (Figure 5b) [77].

Cellulose is a linear polymer formed by residues of D-glucose linked by β-1,4-glycosidic bonds. This is the most abundant and the least complex polysaccharide of the plant cell wall and is degraded by three classes of enzymes, β-1,4-endoglucanases, cellobiohydrolases, and β-glucosidases [3,18,77]. Figure 5c exhibits the families of enzymes related to cellulose hydrolysis. The GH3 family (β-glycosidases) had the highest number of genes, ranging from 7 to 10 copies per genome. In general, families of β-1,4-endoglucanases (families GH5_5, GH5_22, GH9, GH12, GH45, GH131), exoglucanases/cellobiohydrolases (families GH6 and GH7), and β-glucosidases (GH1 and GH3 families) were identified in all fungi. The genes encoding the GH9 and GH45 families were absent in *T. ljubarskyi*. Likewise, in *T. pubescens* and *T. versicolor,* the GH45 gene family was not found (Figure 5c). Finally, 15 to 19 AA9-encoding genes (LPMOs) were identified in the *Trametes* spp. genomes (Figure 5a). AA9 genes are classified as copper-dependent lytic polysaccharide monooxygenases (LPMOs) that act in the oxidative depolymerization of crystalline cellulose [75].

Figure 5d displays the genes encoding enzymes with activity in pectin degradation. As evidenced, the GH28 family, which includes part of the glycosidic hydrolases, especially endo- and exo-polygalacturonases and endo- and exo-rhamnogalacturonases, had the largest number of genes, ranging from 5 to 11 copies per genome. Moreover, other enzymatic families were observed to be involved in pectin hydrolysis, but in a smaller proportion, such as GH78 (two to three genes), GH88 (one gene), GH105 (one to two genes), and GH53 (one gene), which include α-rhamnosidases, unsaturated glucuronyl hydrolases, unsaturated rhamnogalacturone hydrolases, and β-endogalactanases, respectively. In all genomes, pectinmethylesterases (CE8) were identified, and in most of them, enzymes belonging to the CE12 family (pectin acetylesterase) were not found. Finally, it was observed that all *Trametes* spp. genomes contain one gene encoding PL4 (rhamnogalacturonan endolyase), except for *T. polyzona*, which has three genes encoding this enzymatic family.

From the exploratory analysis of CAZymes, a set of genes encoding cellulases, hemicellulases, pectinases, and lignin-modifying enzymes were identified in the genomes of the *Trametes* species. These enzymes act synergistically, contributing to the breakdown of all polymers that make up the plant cell wall [3,7,18,27,77]. Among the analyzed genomes, *T. villosa* CCMB561 was the species with the highest number of genes encoding lignin-modifying enzymes (91 genes) and pectinases (21 genes) and the second with the highest number of genes encoding cellulases (31 genes) and hemicellulases (45 genes). It is also worth mentioning that *T. villosa* CCMB561 harbors all 40 searched genes related to the lignocellulose breakdown. Therefore, this isolate has great potential to be applied in the bioconversion of lignocellulosic biomass in the industry.

## 4. Conclusions

In this study, we demonstrated that through the hybrid assembly using short (Illumina HiSeq) and long reads (Oxford Nanopore MinION), and the assembly workflow MaSuRCA-Purge_dups, a high-quality genome for the isolate *T. villosa* CCMB561 was obtained. The contiguity and completeness of the genome assembled and presented in this study significantly increased when compared to the preliminary and draft version of this isolate previously sequenced using only short reads (Illumina HiSeq). The accurate annotation of the new genome, the comparative genomic analyses, associated with the functional annotation of the CAZymes-encoding genes demonstrated the genetic potential of the isolate *T. villosa* CCMB561 to act in the degradation of all components of lignocellulose. Among the analyzed genomes, *T. villosa* was the species with the highest number of genes encoding lignin-modifying enzymes. Lignin is the most recalcitrant polymer of the plant cell wall and, thus, its removal is considered the most limiting step for the conversion of lignocellulosic biomass. Taken together, data generated in this study provide support for future studies using genomics, transcriptomics, and proteomics tools. Still, they contribute to the understanding of the complex mechanisms involved in the expression, regulation, and evolution of genes and proteins associated with lignocellulose breakdown.

## Figures and Tables

**Table 1:** Summary of the Illumina HiSeq and Oxford Nanopore MinION reads statistics after preprocessing step.

	Illumina	MinION
Total reads number	48,347,940	1,043,247
Total reads bases (bp)	5,798,237,268	4,189,223,607
Coverage	129×	93×
Longest read (bp)	151	21,613
Mean reads length (bp)	138	4476
GC content (%)	57.5	56

**Table 2:** Summary statistics for the assembled genomes of *Trametes villosa* CCMB561 using reads from Illumina HiSeq and Oxford Nanopore MinION.

	Assembly Short Reads (Illumina)	Assembly Oxford Nanopore (MinION)				Hybrid Assembly (Illumina and Oxford Nanopore)		
Assembly/ Software	MaSuRCa	CANU	CANU-smartdenovo	RACON	FLYE	SPADES	MaSuRCa	MaSuRCa-Purge_Dups
Number of contigs (≥0 bp)	4026	1836	337	1836	882	12,829	264	143
Number of contigs (≥500 bp)	3930	1836	337	1836	881	1940	264	143
Largest contig	470,636	1,594,329	1,660,310	1,605,280	1,891,910	1,207,893	4,772,416	9,749,168
Total length (≥500 bp)	58,820,861	63,704,316	42,774,667	63,971,542	49,876,064	65,406,907	62,711,988	46,748,415
GC (%)	59.40	59.36	59.39	59.41	59.35	59.39	59.39	59.45
N50	27,657	103,641	238,816	104,325	204,679	282,055	598,690	1,009,271
L50	503	115	43	114	55	69	21	8
# N’s per 100 kbp	0.00	0.00	0.00	0.00	2.41	227.07	0.16	0.69

**Table 3:** Completeness assessment of *Trametes villosa* CCMB561 assemblies using BUSCO software.

	Complete (%)	Single-Copy (%)	Duplicated (%)	Fragmented (%)	Missing (%)
CANU	80.7	65.2	15.5	6.7	12.6
CANU-smartdenovo	76.2	73.8	2.4	8.6	15.2
FLYE	90.2	85.2	5.0	3.9	5.9
MaSuRCa (Hybrid)	99.0	64.0	35.0	0.2	0.8
MaSuRCa (Illumina)	97.4	64..3	33.1	0.9	1.7
MaSuRCa-Purge_Dups	99.1	96.7	2.4	0.1	0.8
RACON	88.2	70.0	18.2	4.5	7.3
SPADES	99.1	41.6	57.5	0.2	0.7

**Table 4:** Transposable elements (TE) identified in the *Trametes* species.

ID Fungo	Total No. TE	Total TE Coverage%	Retroelements				DNA Transposons	Helitron	Unclassified
			SINEs	LINEs	LTR Elements				
					Ty1/Copia	Gypsy/DIRS1			
*Trametes cinnabarina*	252	8.57	0	144	337	450	390	0	3079
*Trametes coccinea*	104	2.22	0	0	119	137	0	0	1187
*Trametes elegans*	129	4.50	0	0	210	451	14	0	1841
*Trametes hirsuta*	191	10.61	0	73	264	387	65	42	1949
*Trametes ljubarskyi*	172	3.67	0	0	317	263	89	0	2219
*Trametes polyzona*	349	6.41	0	113	416	912	105	69	5043
*Trametes pubescens*	303	5.82	19	106	107	160	258	30	3385
*Trametes sanguinea*	191	7.73	0	41	184	328	32	0	1855
*Trametes versicolor*	234	4.6	0	50	38	144	11	94	4165
*Trametes villosa*	274	7.13	17	97	186	503	74	70	4437

## Data Availability

This Whole Genome Shotgun project has been deposited at DDBJ/ENA/GenBank under the accession PUDQ00000000. The version described in this paper is version PUDQ02000000 (BioSample ID: SAMN08579176, BioProject ID: PRJNA435407).

## Supplementary Materials

The following supporting information can be downloaded at https://www.mdpi.com/article/10.3390/jof8020142/s1, Figure S1: Workflow used for the genome sequencing and assembly of *Trametes villosa* CCMB561. Figure S2: Gene and protein annotation pipeline used for all *Trametes* species. Table S1: Gene annotation statistics generated by GAG software for all genomes from the *Trametes* genus analyzed in our study. Supplementary Data S1: GenomeScope results for the *Trametes villosa* CCMB561. Supplementary Data S2: Annotation of Secondary metabolite gene clusters (SMGCs) of *Trametes villosa* CCMB561. Supplementary Data S3: Annotation of Carbohydrate-active enzymes (CAZymes) for the isolate *Trametes villosa* CCMB561.

## References

[1] Srivastava N., Rawat R., Singh Oberoi H., Ramteke P.W. (2015). A Review on Fuel Ethanol Production from Lignocellulosic Biomass. Int. J. Green Energy.

[2] Isikgor F.H., Becer C.R. (2015). Lignocellulosic biomass: A sustainable platform for the production of bio-based chemicals and polymers. Polym. Chem..

[3] Houfani A.A., Anders N., Spiess A.C., Baldrian P., Benallaoua S. (2020). Insights from enzymatic degradation of cellulose and hemicellulose to fermentable sugars—A review. Biomass Bioenergy.

[4] Hernández-Beltrán J.U., Hernández-De Lira I.O., Cruz-Santos M.M., Saucedo-Luevanos A., Hernández-Terán F., Balagurusamy N. (2019). Insight into Pretreatment Methods of Lignocellulosic Biomass to Increase Biogas Yield: Current State, Challenges, and Opportunities. Appl. Sci..

[5] Bilal M., Nawaz M.Z., Iqbal H.M.N., Hou J., Mahboob S., Al-Ghanim K.A., Cheng H. (2018). Engineering Ligninolytic Consortium for Bioconversion of Lignocelluloses to Ethanol and Chemicals. Protein Pept. Lett..

[6] Liu Y., Wu Y., Zhang Y., Yang X., Yang E., Xu H., Yang Q., Chagan I., Cui X., Chen W. (2019). Lignin degradation potential and draft genome sequence of *Trametes trogii* S0301. Biotechnol. Biofuels.

[7] Floudas D., Binder M., Riley R., Barry K., Blanchette R.A., Henrissat B., Martínez A.T., Otillar R., Spatafora J.W., Yadav J.S. (2012). The Paleozoic Origin of Enzymatic Lignin Decomposition Reconstructed from 31 Fungal Genomes. Science.

[8] Andriani A., Maharani A., Yanto D.H.Y., Pratiwi H., Astuti D., Nuryana I., Agustriana E., Anita S.H., Juanssilfero A.B., Perwitasari U. (2020). Sequential production of ligninolytic, xylanolytic, and cellulolytic enzymes by *Trametes hirsuta* AA-017 under different biomass of Indonesian sorghum accessions-induced cultures. Bioresour. Technol. Rep..

[9] Vasina D.V., Moiseenko K.V., Fedorova T.V., Tyazhelova T.V. (2017). Lignin-degrading peroxidases in white-rot fungus *Trametes hirsuta* 072. Absolute expression quantification of full multigene family. PLoS ONE.

[10] Mäkinen M., Kuuskeri J., Laine P., Smolander O.-P., Kovalchuk A., Zeng Z., Asiegbu F.O., Paulin L., Auvinen P., Lundell T. (2019). Genome description of *Phlebia radiata* 79 with comparative genomics analysis on lignocellulose decomposition machinery of phlebioid fungi. BMC Genom..

[11] Atilano-Camino M.M., Álvarez-Valencia L.H., García-González A., García-Reyes R.B. (2020). Improving laccase production from *Trametes versicolor* using lignocellulosic residues as cosubstrates and evaluation of enzymes for blue wastewater biodegradation. J. Environ. Manag..

[12] Paës G., Navarro D., Benoit Y., Blanquet S., Chabbert B., Chaussepied B., Coutinho P.M., Durand S., Grigoriev I.V., Haon M. (2019). Tracking of enzymatic biomass deconstruction by fungal secretomes highlights markers of lignocellulose recalcitrance. Biotechnol. Biofuels.

[13] Espósito E., Azevedo J.L. (2004). De Fungos—Uma Introdução à Biologia, Bioquímica e Biotecnologia.

[14] Lombard V., Golaconda Ramulu H., Drula E., Coutinho P.M., Henrissat B. (2014). The carbohydrate-active enzymes database (CAZy) in 2013. Nucleic Acids Res..

[15] Cantarel B.L., Coutinho P.M., Rancurel C., Bernard T., Lombard V., Henrissat B. (2009). The Carbohydrate-Active EnZymes database (CAZy): An expert resource for Glycogenomics. Nucleic Acids Res..

[16] Kumar A., Chandra R. (2020). Ligninolytic enzymes and its mechanisms for degradation of lignocellulosic waste in environment. Heliyon.

[17] Mendonça Maciel M.J., Castro e Silva A., Telles Ribeiro H.C. (2010). Industrial and biotechnological applications of ligninolytic enzymes of the basidiomycota: A review. Electron. J. Biotechnol..

[18] Sista Kameshwar A.K., Qin W. (2018). Comparative study of genome-wide plant biomass-degrading CAZymes in white rot, brown rot and soft rot fungi. Mycology.

[19] Daniel G. (2014). Fungal and Bacterial Biodegradation: White Rots, Brown Rots, Soft Rots, and Bacteria. In: Deterioration and protection of sustainable biomaterials. Am. Chem. Soc..

[20] Giweta M. (2020). Role of litter production and its decomposition, and factors affecting the processes in a tropical forest ecosystem: A review. J. Ecol. Environ..

[21] Hage H., Miyauchi S., Virágh M., Drula E., Min B., Chaduli D., Navarro D., Favel A., Norest M., Lesage-Meessen L. (2021). Gene family expansions and transcriptome signatures uncover fungal adaptations to wood decay. Environ. Microbiol..

[22] Couturier M., Navarro D., Chevret D., Henrissat B., Piumi F., Ruiz-Dueñas F.J., Martinez A.T., Grigoriev I.V., Riley R., Lipzen A. (2015). Enhanced degradation of softwood versus hardwood by the white-rot fungus *Pycnoporus coccineus*. Biotechnol. Biofuels.

[23] Pavlov A.R., Tyazhelova T.V., Moiseenko K.V., Vasina D.V., Mosunova O.V., Fedorova T.V., Maloshenok L.G., Landesman E.O., Bruskin S.A., Psurtseva N.V. (2015). Draft Genome Sequence of the Fungus *Trametes hirsuta* 072. Genome Announc..

[24] Ferreira D.S.S., Kato R.B., Miranda F.M., da Costa Pinheiro K., Fonseca P.L.C., Tomé L.M.R., Vaz A.B.M., Badotti F., Ramos R.T.J., Brenig B. (2018). Draft genome sequence of *Trametes villosa* (Sw.) Kreisel CCMB561, a tropical white-rot Basidiomycota from the semiarid region of Brazil. Data Br..

[25] Busk P.K., Lange M., Pilgaard B., Lange L. (2014). Several Genes Encoding Enzymes with the Same Activity Are Necessary for Aerobic Fungal Degradation of Cellulose in Nature. PLoS ONE.

[26] Granchi Z., Peng M., Chi-A-Woeng T., de Vries R.P., Hildén K., Mäkelä M.R. (2017). Genome Sequence of the Basidiomycete White-Rot Fungus *Trametes pubescens* FBCC735. Genome Announc..

[27] Miyauchi S., Rancon A., Drula E., Hage H., Chaduli D., Favel A., Grisel S., Henrissat B., Herpoël-Gimbert I., Ruiz-Dueñas F.J. (2018). Integrative visual omics of the white-rot fungus *Polyporus brumalis* exposes the biotechnological potential of its oxidative enzymes for delignifying raw plant biomass. Biotechnol. Biofuels.

[28] Lin W., Jia G., Sun H., Sun T., Hou D. (2020). Genome sequence of the fungus *Pycnoporus sanguineus*, which produces cinnabarinic acid and pH- and thermo- stable laccases. Gene.

[29] Carneiro R.T., Lopes M.A., Silva M.L., Santos V.D., Souza V.B., Sousa A.O., Pirovani C.P., Koblitz M.G., Benevides R.G., Góes-Neto A. (2017). *Trametes villosa* Lignin Peroxidase (TvLiP): Genetic and Molecular Characterization. J. Microbiol. Biotechnol..

[30] Coniglio R.O., Díaz G.V., Fonseca M.I., Castrillo M.L., Piccinni F.E., Villalba L.L., Campos E., Zapata P.D. (2020). Enzymatic hydrolysis of barley straw for biofuel industry using a novel strain of *Trametes villosa* from Paranaense rainforest. Prep. Biochem. Biotechnol..

[31] Yamanaka R., Soares C.F., Matheus D.R., Machado K.M.G. (2008). Lignolytic enzymes produced by *Trametes villosa* ccb176 under different culture conditions. Braz. J. Microbiol..

[32] Silva M.L., de Souza V.B., da Silva Santos V., Kamida H.M., de Vasconcellos-Neto J.R., Góes-Neto A., Koblitz M.G. (2014). Production of Manganese Peroxidase by *Trametes villosa* on Unexpensive Substrate and Its Application in the Removal of Lignin from Agricultural Wastes. Adv. Biosci. Biotechnol..

[33] White T.J., Bruns T., Lee S., Taylor J. (1990). Amplification and Direct Sequencing of Fungal Ribosomal RNA Genes for Phylogenetics. PCR Protocols.

[34] Tomé L.M.R., Badotti F., Assis G.B.N., Fonseca P.L.C., da Silva G.A., da Silveira R.M.B., Costa-Rezende D.H., dos Santos E.R.D., de Carvalho Azevedo V.A., Figueiredo H.C.P. (2019). Proteomic fingerprinting for the fast and accurate identification of species in the Polyporoid and Hymenochaetoid fungi clades. J. Proteom..

[35] Marçais G., Kingsford C. (2011). A fast, lock-free approach for efficient parallel counting of occurrences of k-mers. Bioinformatics.

[36] Ranallo-Benavidez T.R., Jaron K.S., Schatz M.C. (2020). GenomeScope 2.0 and Smudgeplot for reference-free profiling of polyploid genomes. Nat. Commun..

[37] Kolmogorov M., Yuan J., Lin Y., Pevzner P.A. (2019). Assembly of long, error-prone reads using repeat graphs. Nat. Biotechnol..

[38] Koren S., Walenz B.P., Berlin K., Miller J.R., Bergman N.H., Phillippy A.M. (2017). Canu: Scalable and accurate long-read assembly via adaptive k -mer weighting and repeat separation. Genome Res..

[39] Vaser R., Sović I., Nagarajan N., Šikić M. (2017). Fast and accurate de novo genome assembly from long uncorrected reads. Genome Res..

[40] Schmidt M.H.-W., Vogel A., Denton A.K., Istace B., Wormit A., van de Geest H., Bolger M.E., Alseekh S., Maß J., Pfaff C. (2017). De Novo Assembly of a New *Solanum pennellii* Accession Using Nanopore Sequencing. Plant Cell.

[41] Zimin A.V., Marçais G., Puiu D., Roberts M., Salzberg S.L., Yorke J.A. (2013). The MaSuRCA genome assembler. Bioinformatics.

[42] Bankevich A., Nurk S., Antipov D., Gurevich A.A., Dvorkin M., Kulikov A.S., Lesin V.M., Nikolenko S.I., Pham S., Prjibelski A.D. (2012). SPAdes: A New Genome Assembly Algorithm and Its Applications to Single-Cell Sequencing. J. Comput. Biol..

[43] Guan D., McCarthy S.A., Wood J., Howe K., Wang Y., Durbin R. (2020). Identifying and removing haplotypic duplication in primary genome assemblies. Bioinformatics.

[44] Gurevich A., Saveliev V., Vyahhi N., Tesler G. (2013). QUAST: Quality assessment tool for genome assemblies. Bioinformatics.

[45] Simão F.A., Waterhouse R.M., Ioannidis P., Kriventseva E.V., Zdobnov E.M. (2015). BUSCO: Assessing genome assembly and annotation completeness with single-copy orthologs. Bioinformatics.

[46] Cantarel B.L., Korf I., Robb S.M.C., Parra G., Ross E., Moore B., Holt C., Sanchez Alvarado A., Yandell M. (2007). MAKER: An easy-to-use annotation pipeline designed for emerging model organism genomes. Genome Res..

[47] Holt C., Yandell M. (2011). MAKER2: An annotation pipeline and genome-database management tool for second-generation genome projects. BMC Bioinform..

[48] Campbell M.S., Holt C., Moore B., Yandell M. (2014). Genome Annotation and Curation Using MAKER and MAKER-P. Curr. Protoc. Bioinform..

[49] Korf I. (2004). Gene finding in novel genomes. BMC Bioinform..

[50] Stanke M., Steinkamp R., Waack S., Morgenstern B. (2004). AUGUSTUS: A web server for gene finding in eukaryotes. Nucleic Acids Res..

[51] Lomsadze A. (2005). Gene identification in novel eukaryotic genomes by self-training algorithm. Nucleic Acids Res..

[52] Tarailo-Graovac M., Chen N. (2009). Using Repeat Masker to Identify Repetitive Elements in Genomic Sequences. Curr. Protoc. Bioinform..

[53] Geib S.M., Hall B., Derego T., Bremer F.T., Cannoles K., Sim S.B. (2018). Genome Annotation Generator: A simple tool for generating and correcting WGS annotation tables for NCBI submission. Gigascience.

[54] Araujo F.A., Barh D., Silva A., Guimarães L., Ramos R.T.J. (2018). GO FEAT: A rapid web-based functional annotation tool for genomic and transcriptomic data. Sci. Rep..

[55] Lowe T.M., Eddy S.R. (1997). tRNAscan-SE: A Program for Improved Detection of Transfer RNA Genes in Genomic Sequence. Nucleic Acids Res..

[56] Blin K., Shaw S., Kloosterman A.M., Charlop-Powers Z., van Wezel G.P., Medema M.H., Weber T. (2021). antiSMASH 6.0: Improving cluster detection and comparison capabilities. Nucleic Acids Res..

[57] Bao Z. (2002). Automated De Novo Identification of Repeat Sequence Families in Sequenced Genomes. Genome Res..

[58] Price A.L., Jones N.C., Pevzner P.A. (2005). De novo identification of repeat families in large genomes. Bioinformatics.

[59] Benson G. (1999). Tandem repeats finder: A program to analyze DNA sequences. Nucleic Acids Res..

[60] Capella-Gutierrez S., Silla-Martinez J.M., Gabaldon T. (2009). trimAl: A tool for automated alignment trimming in large-scale phylogenetic analyses. Bioinformatics.

[61] Nguyen L.-T., Schmidt H.A., von Haeseler A., Minh B.Q. (2015). IQ-TREE: A Fast and Effective Stochastic Algorithm for Estimating Maximum-Likelihood Phylogenies. Mol. Biol. Evol..

[62] Zhang H., Yohe T., Huang L., Entwistle S., Wu P., Yang Z., Busk P.K., Xu Y., Yin Y. (2018). dbCAN2: A meta server for automated carbohydrate-active enzyme annotation. Nucleic Acids Res..

[63] Dutreux F., Da Silva C., D’Agata L., Couloux A., Gay E.J., Istace B., Lapalu N., Lemainque A., Linglin J., Noel B. (2018). De novo assembly and annotation of three *Leptosphaeria* genomes using Oxford Nanopore MinION sequencing. Sci. Data.

[64] Minei R., Hoshina R., Ogura A. (2018). De novo assembly of middle-sized genome using MinION and Illumina sequencers. BMC Genom..

[65] De Carvalho L.M., Borelli G., Camargo A.P., de Assis M.A., de Ferraz S.M.F., Fiamenghi M.B., José J., Mofatto L.S., Nagamatsu S.T., Persinoti G.F. (2019). Bioinformatics applied to biotechnology: A review towards bioenergy research. Biomass Bioenergy.

[66] Maggiori C., Raymond-Bouchard I., Brennan L., Touchette D., Whyte L. (2021). MinION sequencing from sea ice cryoconites leads to de novo genome reconstruction from metagenomes. Sci. Rep..

[67] Miyauchi S., Navarro D., Grigoriev I.V., Lipzen A., Riley R., Chevret D., Grisel S., Berrin J.-G., Henrissat B., Rosso M.-N. (2016). Visual Comparative Omics of Fungi for Plant Biomass Deconstruction. Front. Microbiol..

[68] Sun X., He C., Fang Z., Xiao Y. (2018). Expression and characterization of NADPH-cytochrome P450 reductase from *Trametes versicolor* in *Escherichia coli*. Sheng Wu Gong Cheng Xue Bao.

[69] Chen W., Lee M.-K., Jefcoate C., Kim S.-C., Chen F., Yu J.-H. (2014). Fungal Cytochrome P450 Monooxygenases: Their Distribution, Structure, Functions, Family Expansion, and Evolutionary Origin. Genome Biol. Evol..

[70] Wang M., Ruan R., Li H. (2021). The completed genome sequence of the pathogenic ascomycete fungus *Penicillium digitatum*. Genomics.

[71] Osbourn A. (2010). Secondary metabolic gene clusters: Evolutionary toolkits for chemical innovation. Trends Genet..

[72] Xiao H., Zhong J.-J. (2016). Production of Useful Terpenoids by Higher-Fungus Cell Factory and Synthetic Biology Approaches. Trends Biotechnol..

[73] Brandenburger E., Gressler M., Leonhardt R., Lackner G., Habel A., Hertweck C., Brock M., Hoffmeister D. (2017). A Highly Conserved Basidiomycete Peptide Synthetase Produces a Trimeric Hydroxamate Siderophore. Appl. Environ. Microbiol..

[74] Miethke M., Marahiel M.A. (2007). Siderophore-Based Iron Acquisition and Pathogen Control. Microbiol. Mol. Biol. Rev..

[75] Levasseur A., Drula E., Lombard V., Coutinho P.M., Henrissat B. (2013). Expansion of the enzymatic repertoire of the CAZy database to integrate auxiliary redox enzymes. Biotechnol. Biofuels.

[76] Sidar A., Albuquerque E.D., Voshol G.P., Ram A.F.J., Vijgenboom E., Punt P.J. (2020). Carbohydrate Binding Modules: Diversity of Domain Architecture in Amylases and Cellulases from Filamentous Microorganisms. Front. Bioeng. Biotechnol..

[77] Van den Brink J., de Vries R.P. (2011). Fungal enzyme sets for plant polysaccharide degradation. Appl. Microbiol. Biotechnol..

[78] Justo A., Hibbett D.S. (2011). Phylogenetic classification of *Trametes* (Basidiomycota, Polyporales) based on a five-marker dataset. Taxon.

[79] Mohanta T.K., Bae H. (2015). The diversity of fungal genome. Biol. Proced. Online.

[80] Muszewska A., Steczkiewicz K., Stepniewska-Dziubinska M., Ginalski K. (2017). Cut-and-Paste Transposons in Fungi with Diverse Lifestyles. Genome Biol. Evol..

[81] Muszewska A., Steczkiewicz K., Stepniewska-Dziubinska M., Ginalski K. (2019). Transposable elements contribute to fungal genes and impact fungal lifestyle. Sci. Rep..

[82] Castanera R., Borgognone A., Pisabarro A.G., Ramírez L. (2017). Biology, dynamics, and applications of transposable elements in basidiomycete fungi. Appl. Microbiol. Biotechnol..

[83] Raina M., Ibba M. (2014). tRNAs as regulators of biological processes. Front. Genet..

[84] Stajich J.E. (2017). Fungal genomes and insights into the evolution of the kingdom. Microbiol. Spectr..

[85] Wong D.W.S. (2009). Structure and Action Mechanism of Ligninolytic Enzymes. Appl. Biochem. Biotechnol..

[86] Dashtban M., Schraft H., Syed T.A., Qin W. (2010). Fungal biodegradation and enzymatic modification of lignin. Int. J. Biochem. Mol. Biol..

[87] Couturier M., Ladevèze S., Sulzenbacher G., Ciano L., Fanuel M., Moreau C., Villares A., Cathala B., Chaspoul F., Frandsen K.E. (2018). Lytic xylan oxidases from wood-decay fungi unlock biomass degradation. Nat. Chem. Biol..

