# Supplementary material for: Hybrid Assembly Improves Genome Quality and Completeness of Trametes villosa CCMB561 and Reveals a Huge Potential for Lignocellulose Breakdown
Source: J Fungi (Basel). 2022 Jan 30;8(2):142. doi: 10.3390/jof8020142 (PMC8876698; doi:10.3390/jof8020142)
Supplement: Supplementary file 1 [file jof-08-00142-s001.zip › Figure S2.pdf]

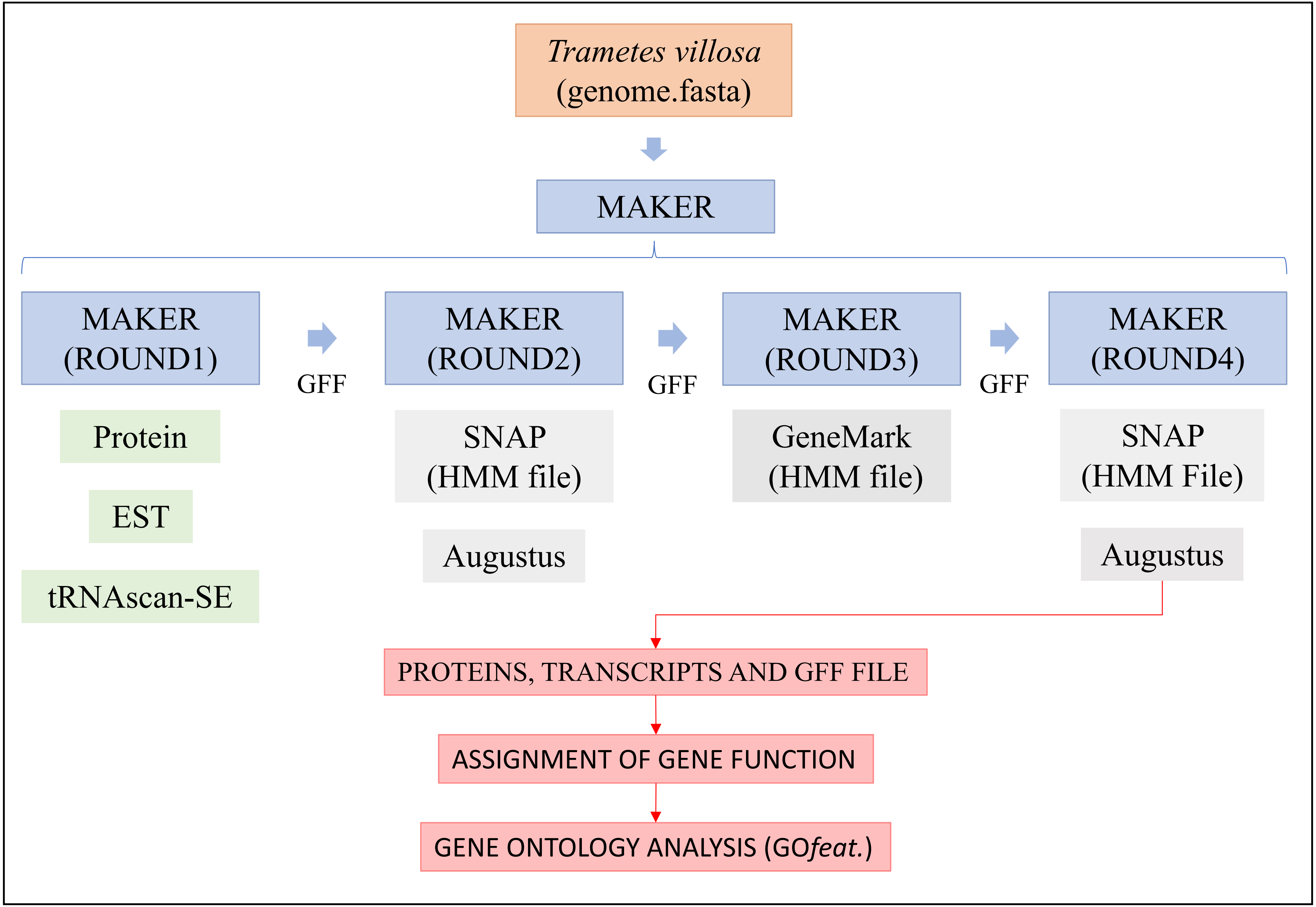

Trametes villosa
(genome.fasta)
MAKER
MAKER
(ROUND1)
GFF
MAKER
(ROUND2)
GFF
MAKER
(ROUND3)
GFF
MAKER
(ROUND4)
Protein
EST
tRNAscan-SE
SNAP
(HMM file)
Augustus
GeneMark
(HMM file)
SNAP
(HMM File)
Augustus
PROTEINS, TRANSCRIPTS AND GFF FILE
ASSIGNMENT OF GENE FUNCTION
GENE ONTOLOGY ANALYSIS (GO*feat.*)
