# Supplementary material for: Hybrid Assembly Improves Genome Quality and Completeness of Trametes villosa CCMB561 and Reveals a Huge Potential for Lignocellulose Breakdown
Source: J Fungi (Basel). 2022 Jan 30;8(2):142. doi: 10.3390/jof8020142 (PMC8876698; doi:10.3390/jof8020142)
Supplement: Supplementary file 1 [file jof-08-00142-s001.zip › Supplementary Data 1.pdf]

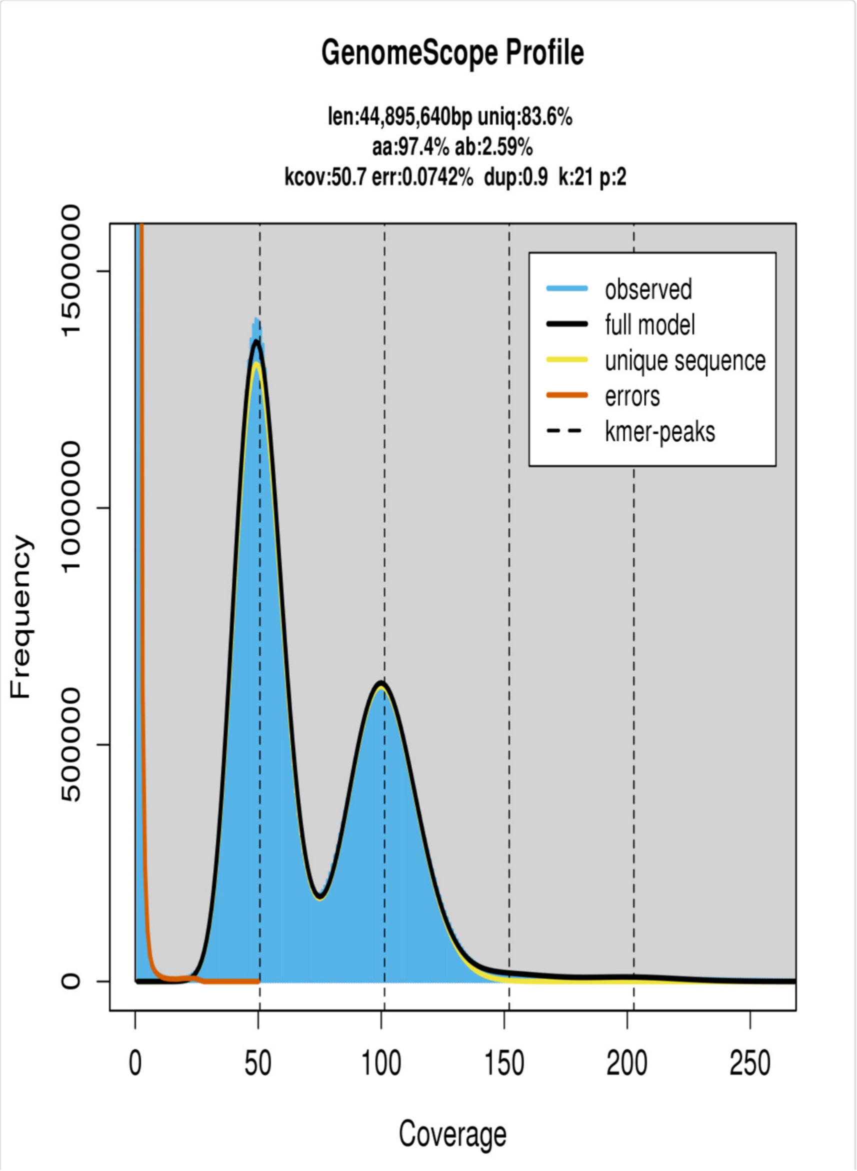
GenomeScope Profile
len:44,895,640bp uniq:83.6%
aa:97.4% ab:2.59%
kcov:50.7 err:0.0742% dup:0.9 k:21 p:2
observed
full model
unique sequence
errors
kmer-peaks
Frequency
0
500000
1000000
1500000
0
50
100
150
200
250
Coverage

```
GenomeScope version 2.0
input file = user_uploads/3ZGtdEQ9yidRAYrvKddc
output directory = user_data/3ZGtdEQ9yidRAYrvKddc
p = 2
k = 21

property                      min               max
Homozygous (aa)               97.3963%          97.4139%
Heterozygous (ab)             2.58608%          2.60366%
Genome Haploid Length         44,851,312 bp     44,895,640 bp
Genome Repeat Length          7,349,356 bp      7,356,619 bp
Genome Unique Length          37,501,956 bp     37,539,020 bp
Model Fit                     85.8514%          95.4174%
Read Error Rate               0.0741602%        0.0741602%
```

Model

```
Formula: y_transform ~ x^transform_exp * length * predict2_0(r1, k, d,
    kmercov, bias, x)

Parameters:
         Estimate Std. Error t value Pr(>|t|)
d       3.485e-02  8.608e-04   40.48   <2e-16 ***
r1      2.595e-02  4.396e-05  590.34   <2e-16 ***
kmercov 5.067e+01  1.251e-02 4049.23   <2e-16 ***
bias    9.004e-01  5.090e-03  176.89   <2e-16 ***
length  3.888e+07  4.836e+04  803.84   <2e-16 ***
---
Signif. codes:  0 '***' 0.001 '**' 0.01 '*' 0.05 '.' 0.1 ' ' 1

Residual standard error: 448900 on 1995 degrees of freedom

Number of iterations to convergence: 6
Achieved convergence tolerance: 1.49e-08
```
