# Supplementary material for: Hybrid Assembly Improves Genome Quality and Completeness of Trametes villosa CCMB561 and Reveals a Huge Potential for Lignocellulose Breakdown
Source: J Fungi (Basel). 2022 Jan 30;8(2):142. doi: 10.3390/jof8020142 (PMC8876698; doi:10.3390/jof8020142)
Supplement: Supplementary file 1 [file jof-08-00142-s001.zip › Table S1.pdf]

Table S1 – Gene annotation statistics done by GAG software for all genomes of *Trametes* annotated.

	Trametes villosa	*Trametes versicolor*	*Trametes pubescens*	*Trametes polyzona*	*Trametes elegans*	*Trametes hirsuta*	*Trametes ljubarskyi*	*Trametes cinnabarina*	*Trametes sanguinea*	*Trametes coccinea*
Number of genes	14,540	14,172	14,153	12,203	10,725	11,490	10,821	11,069	13,143	11,900
Number of exons	86,516	85,492	80,744	68,461	64,521	68,379	66,939	68,002	79,285	70,306
Number of introns	71,976	71,317	66,588	56,257	53,796	56,888	56,118	56,932	66,142	58,406
Mean gene length	2,121	2,092	1,903	1,779	1,933	2,018	2,003	1,971	1,934	1,920
Mean exon length	265	253	257	246	247	261	248	245	239	249
Mean intron length	112	115	95	88	91	96	92	93	99	94
Mean CDS length	1,572	1,514	1,455	1,379	1,487	1,551	1,535	1,504	1,443	1,469
% of genome covered by genes	66,0	66,2	67,8	59,3	62,7	61,9	63,7	64,8	69,5	69,7
% of genome covered by CDS	48,9	47,9	51,8	45,9	48,2	47,6	48,8	49,5	51,8	53,4
Mean exons per mRNA	6	6	6	6	6	6	6	6	6	6
Mean introns per mRNA	5	5	5	5	5	5	5	5	5	5
