# Supplementary figures and images for: Hybrid Assembly Improves Genome Quality and Completeness of Trametes villosa CCMB561 and Reveals a Huge Potential for Lignocellulose Breakdown
Source: J Fungi (Basel). 2022 Jan 30;8(2):142. doi: 10.3390/jof8020142 (PMC8876698; doi:10.3390/jof8020142)

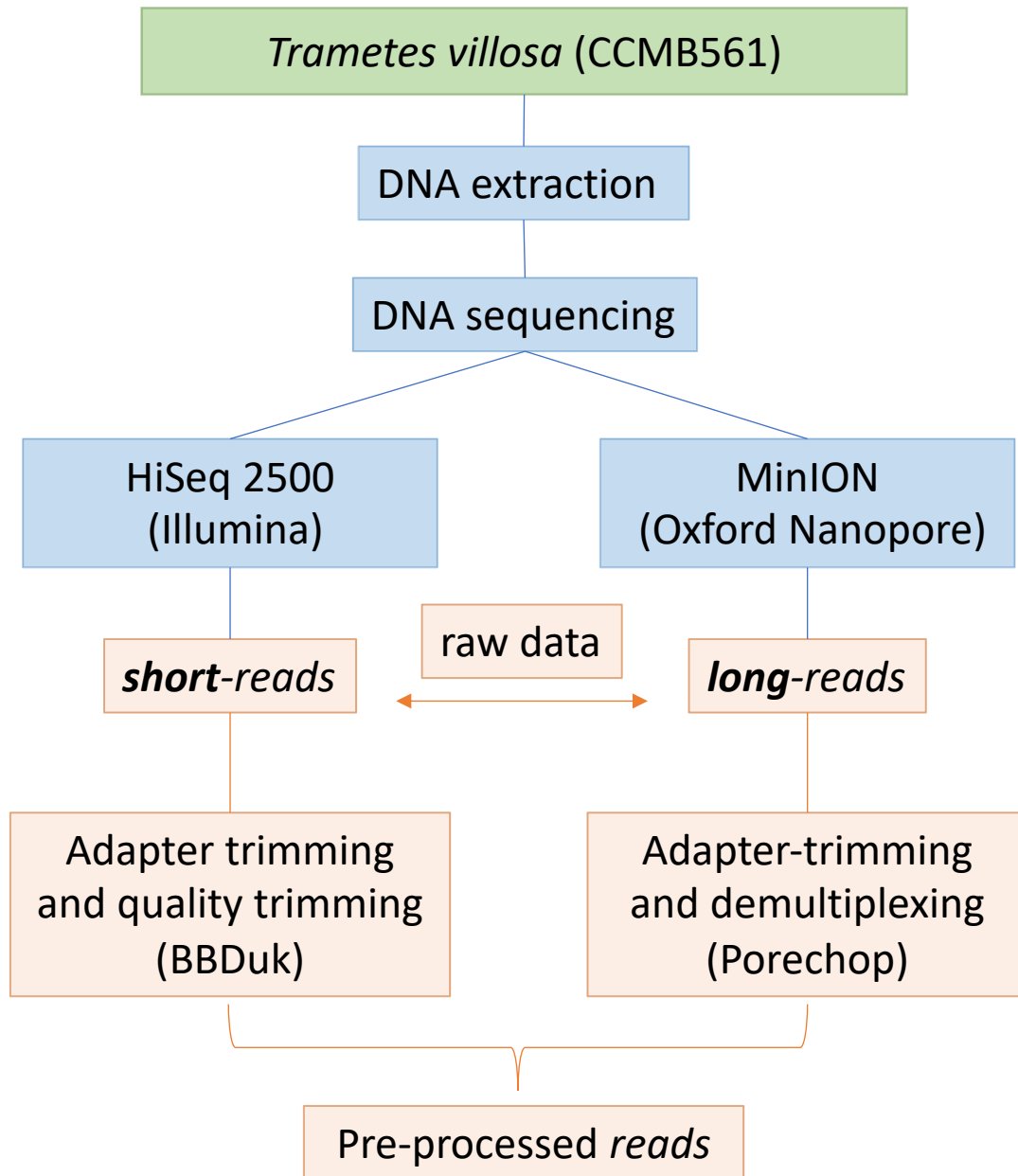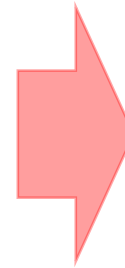

Software used in the assembly

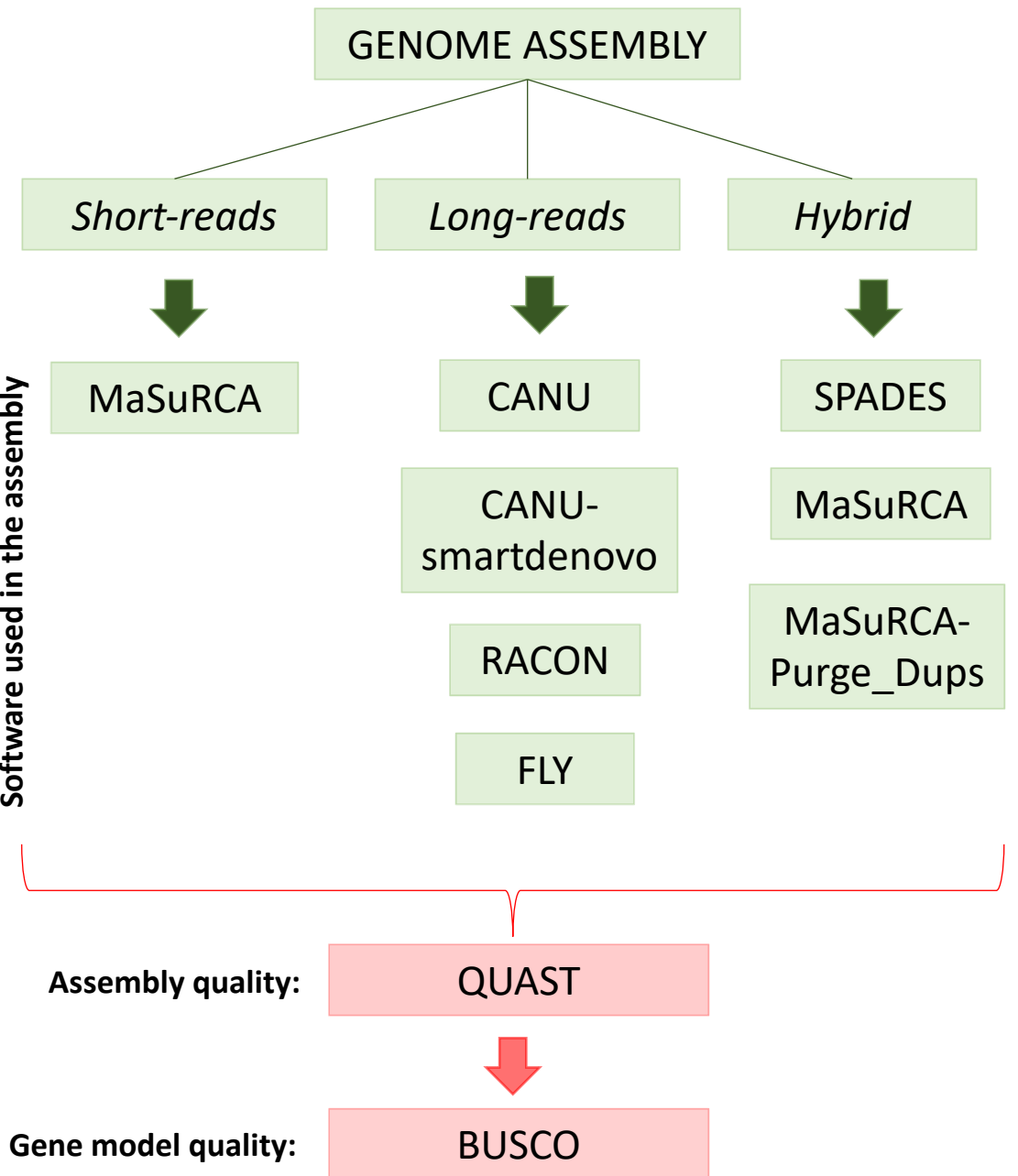

Supplement: Supplementary file 1 [file jof-08-00142-s001.zip › Figure S1.pdf]
